# Intranasal Synechiae as Complications of Rhinosurgical Treatment—A Review of Current Knowledge

**DOI:** 10.3390/jcm12216831

**Published:** 2023-10-29

**Authors:** Mateusz J. Stępiński, Jacek Banaszewski

**Affiliations:** 1 Department of Laryngology with Maxillofacial Surgery Subdepartment, Multidisciplinary Regional Hospital, Dekerta 1, 66-400 Gorzow Wielkopolski, Poland; 2 Department of Otolaryngology and Laryngological Oncology, Poznan University of Medical Sciences, Przybyszewskiego 49, 60-355 Poznan, Poland

**Keywords:** synechia, nasal adhesions, rhinosurgical treatment, complication, FESS, functional endoscopic sinus surgery, septoplasty, nasal mucosa, systematic review

## Abstract

Intranasal adhesions (synechiae) develop as a result of improper healing of the nasal mucosa. Their incidence ranges from 6.8% to 36% of rhinosurgical procedures. The aim of this study was to review the available publications and monographs dealing with intranasal adhesions—both in the context of formation and risk factors. The study used a review of the literature to determine the articles and studies available in the following medical databases: MEDLINE (National Library of Medicine’s), PubMed, and Google Scholar. The following search terms were used: synechiae nasal + synechial nasal + intranasal adhesions + nasal adhesions. The time criterion of available materials was not applied. Available filters in the search engines were used to narrow down the search results. Artificial intelligence was not applied. The review indicated that the risk of intranasal adhesions correlates with the type of surgery, the surgical technique, the dressing materials, and wound care in the postoperative period. Every case requires an individualized approach. Nasal septum separators, (self-)dissolving dressings and (in selected cases) Mitomycin C were investigated thoroughly. Further studies are required which may result in a universal classification system for intranasal adhesions.

## 1. Introduction

**Figure 1:**
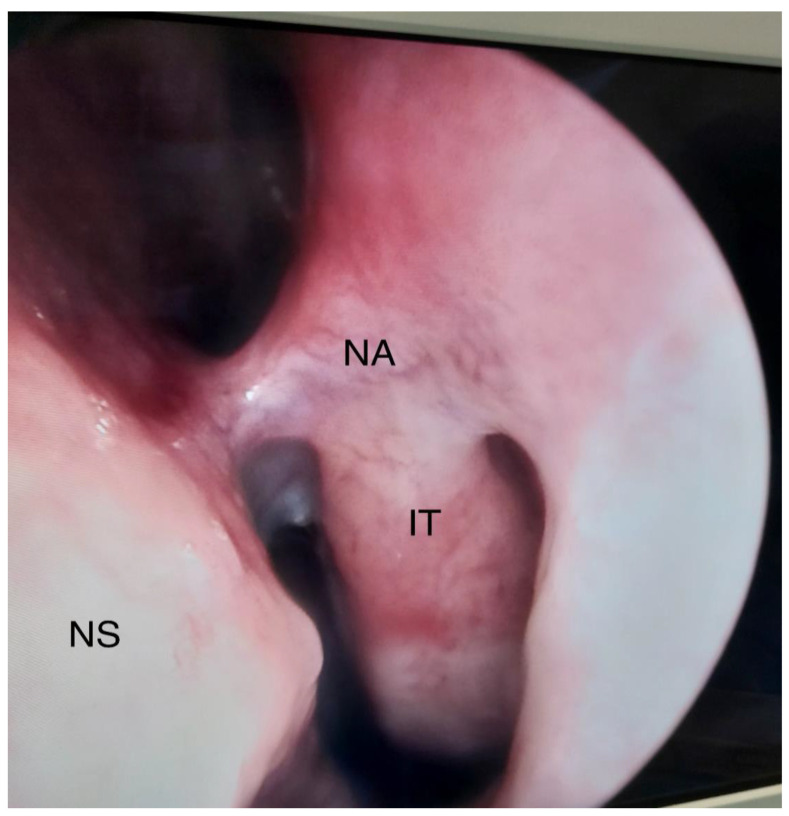
Adhesion between the nasal septum and the inferior turbinate, left side. NS—Nasal septum, IT—inferior turbinate, NA—nasal adhesion.

**Figure 2:**
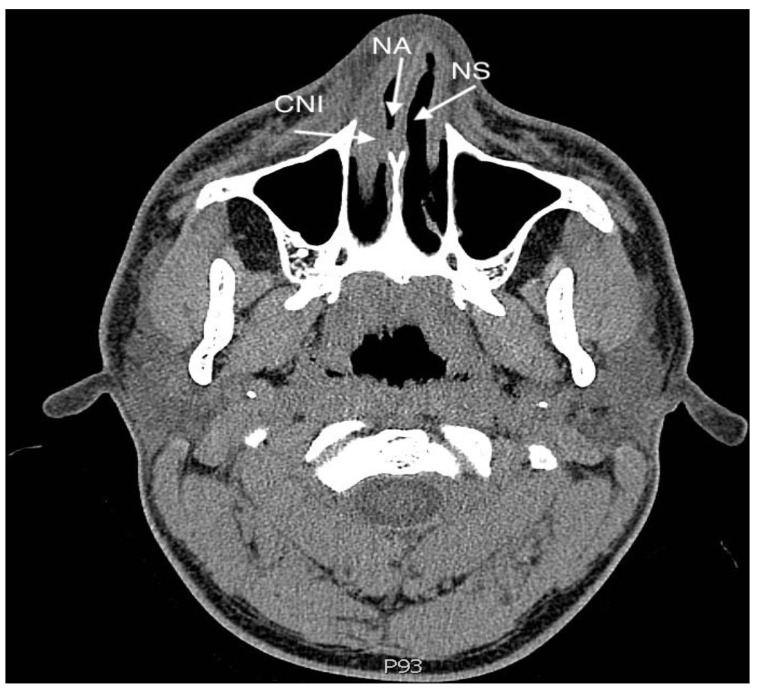
Facial computed tomography scanning of. An adhesion is present between the inferior nasal turbinate and the nasal septum, right side. NS—Nasal septum, CNI—concha nasalis inferior (inferior turbinate), NA—nasal adhesion.

Intranasal adhesions (synechiae) develop as a result of improper healing of the nasal mucosa. The incidence of this complication of rhinosurgical procedures ranges from 6.8% up to 36.2% [1,2]. The issue of intranasal adhesions occurring after operations within the nasal cavity has been addressed by scientists since as early as 1987 and still requires a thorough analysis [3]. The aim of this study was to review the available publications and monographs dealing with intranasal adhesions—both in the context of formation and risk factors (surgical techniques, methods of prevention, and materials used). Figure 1 and Figure 2 show an adhesion between the nasal septum and the inferior turbinate (left side), and facial computed tomography scanning of. An adhesion is present between the inferior nasal turbinate and nasal septum (right side).

## 2. Materials and Methods

The study used a review of the literature to determine the articles and studies available in the following medical databases: MEDLINE (National Library of Medicine’s), PubMed, and Google Scholar. The following search terms were used: synechiae nasal + synechial nasal + intranasal adhesions + nasal adhesions. Reports from 1915 to 31 December 2022 were identified. The following filters available in the search engines were used to narrow down the search results: “Free full text”, “Books and Documents”, “Clinical Trial”, “Meta-Analysis”, “Randomized Controlled Trial”, “Review”, and “Systematic Review”.

The following exclusion criteria were employed: papers with only the abstract available, studies dealing with fields other than rhinosurgery and papers published in languages other than English.

Artificial intelligence was not applied. Duplicate reports and double publications from the same cases were excluded. The authors reviewed the databases together and evaluated individual results. All reports were assessed for eligibility and reviewed by the authors. The full list of included articles can be found in the “Supplementary Materials” section.

## 3. Results

Three databases were reviewed and 1610 papers were identified. Initially, using automatic filtering, case studies, reprints and editorial limitations were excluded (1426). Subsequently, papers with only abstracts available, studies dealing with fields other than rhinosurgery and papers published in languages other than English were excluded (138).

**Figure 3:**
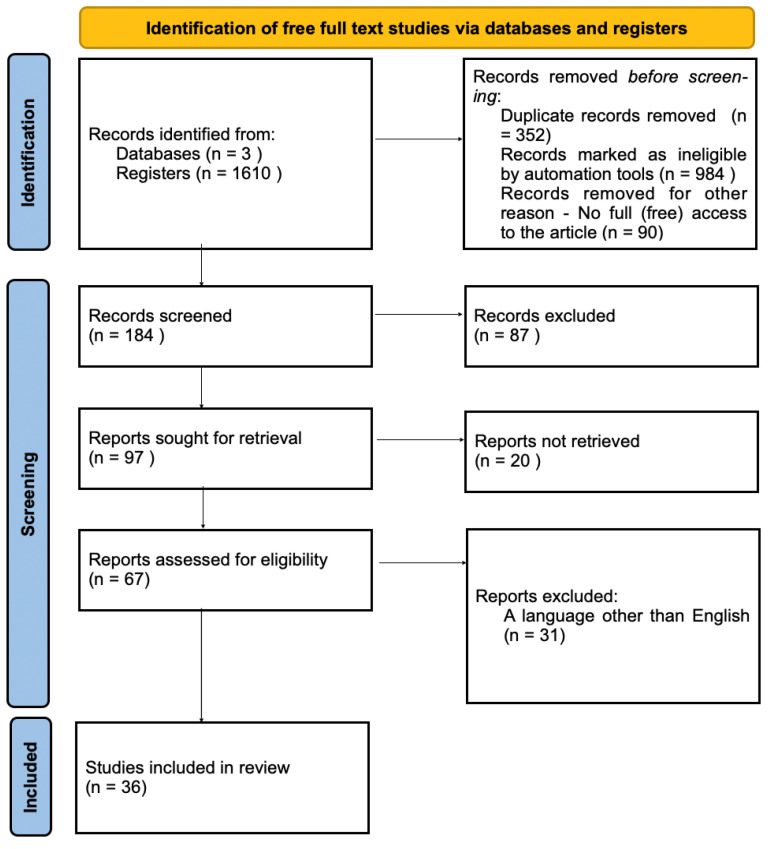
Identification of free full studies via databases.

Thirty-six papers were included in the final stage, including review papers, randomized trials and meta-analyses (Figure 3).

The first reports about the documented treatment of intranasal adhesions date back to 1915. McKenzie described a method of releasing adhesions using diathermy during a procedure performed on a soldier (the adhesions occurred secondary to the pathological healing of the face and nose wound caused by a bullet fragment) [4].

### 3.1. The Use of Nasal Splints (Separators) as a Prevention of Intranasal Adhesions (Synechiae)

The prevention of adhesion formation dates back to 1955, when nasal splits (made of X-ray films) were initially used. Over time, technological progress has led to a number of modifications in terms of the material and the form of separators. Currently, silicone rubber or polytetrafluoroethylene (Teflon) are used [5].

#### 3.1.1. The Use of Nasal Splints in the Surgery of the Inferior Turbinates (Turbinoplasty)

Awad and Hamid conducted a randomized, prospective, double-blind study in a group of approximately 100 patients, evaluating the use of nasal separators (Atos Medical AB, Horby, Sweden) after a bilateral partial resection of the inferior turbinates by classical surgery. The resection involved the mucosa and a part of the inferior turbinate bone (depending on the degree of hypertrophy). The study indicated that the use of nasal separators after partial resections of the inferior turbinates statistically significantly reduces the incidence of intranasal adhesions [6].

#### 3.1.2. The Use of Nasal Splints in Septoturbinoplasty

Tang and Kanker published a study (six randomized trials were analyzed), in which patients after rhinosurgical treatment (surgery of the nasal septum and inferior turbinates) were evaluated. They found that the use of conventional septal separators was not justified since they may cause an increase in pain without a statistically significant reduction in the risk of intranasal adhesions. At the same time, one of the studies, in which thinner separators (made of Silastic) were used, had promising results suggesting a real reduction in postoperative discomfort and improvement in the condition of the nasal mucosa [7,8,9].

Nasal septum separators made of polyethylene (secured with situational sutures) were also investigated. They were held in place by a medical-grade stainless steel spring clip, compared to a rubber glove-based tampon. It was proved that late complications, such as intranasal synechiae, were less frequent in the group of patients with whom separators were used. However, these results were not statistically significant [10].

#### 3.1.3. Nasal Separators Used in Endoscopic Operations of the Paranasal Sinuses

Adhesions occurring after endoscopic sinus surgery (ESS), located at the level of the middle meatus between the middle turbinate and the lateral wall of the nose, also pose significant problems.

Studies indicate a reduction in the incidence of adhesions after endoscopic sinus surgery when Silastic separators are used [11].

Hartl et al. described their original idea of modifying the Silastic splint, placed in the middle meatus after endoscopic sinus surgery, and consisting of the creation of openings to facilitate drainage of the sinuses. In addition to economic benefits, the authors also indicated a reduction in the risk of developing intranasal adhesions. Despite the large (250 people) group subjected to the procedure, the authors did not provide a control group and statistical analysis [12]. The concept of using modifications may have beneficial clinical implications, but requires more careful analysis, preferably with a control group.

### 3.2. The Use of the Suturing of the (Trans)Nasal Septum

An alternative to the use of anterior nasal tamponade (after nasal septum surgery), reducing the level of discomfort during the removal of the dressing material, is to perform a (perforating) suture within the nasal septum [13]. Both the animal model (rabbits) and clinical trials did not show a statistically significant difference in complications (mucosa atrophy and crusting) between the two methods [14,15]. In addition, one of the studies, conducted on a population of 200 people, showed a statistically significant reduction in the formation of intranasal adhesions in cases of nasal septum suture (as opposed to a bandage tamponade impregnated with an antibiotic) [16]. Another study conducted in a larger population (697 patients undergoing septoplasty) showed no statistically significant differences in the development of bleeding complications, or in the formation of nasal septal hematoma, septal perforation, or intranasal adhesions [17].

However, the prolongation of the operation time with the use of nasal septum suturing seems indisputable [18].

### 3.3. Irrigation of the Nasal Cavity and Paranasal Sinuses after Rhinosurgical Treatment

The beneficial effects of irrigation of the nasal cavities and paranasal sinuses in the postoperative period are indisputable. Irrigations help to remove tissues after surgery (including blood clots), accelerate the reconstruction of normal mucosa and improve mucociliary clearance (MCC) [19,20].

In addition, the type and the concentration of the substances used for irrigation are also important.

Kurtaran et al. evaluated patients after nasal septum surgery with bilateral conchoplasty (by radio coagulation), where different concentrations of irrigation solutions were used (tap water, buffered isotonic saline, buffered isotonic salt with xylitol, and hypertonic 2.3% sea water). It has been proven that a 2.3% solution of sodium chloride in the form of hypertonic sea water was the most effective in reducing dryness, swelling, adhesions and nasal obstruction, as well as in evacuating pathological tissue deposits arising after nasal septum surgery with conchoplasty (performed by radio coagulation) [21].

One prospective study on a relatively small (46 subjects) study group evaluated the effects of irrigation with 0.9% sodium chloride in combination with nebulizations of 9 mg of sodium hyaluronate suspended in 3 mL of saline, compared to nebulizations of 6 mL of saline, in patients undergoing endoscopic functional surgery of the paranasal sinuses (FESS). Despite the lack of a statistically significant difference in the incidence of intranasal adhesions, the study showed a beneficial effect of the use of sodium hyaluronate on the process of cilia motility, as well as an improvement in the development of the nasal mucosa and the feeling of patency of the nasal cavities [22].

A study conducted on an animal model (rats) showed that the use of sodium hyaluronate and carboxymethylcellulose (S-HA/CMC) solution statistically significantly reduces the risk of iatrogenic intranasal adhesions. In their conclusions, however, the authors emphasize the need to continue research in clinical conditions, giving hope for an effective alternative to the use of anterior nasal tamponade [23].

A therapeutic option may also be biocompatible, non-toxic substances of natural origin, e.g., chiotosan—a polysaccharide obtained from the shells of marine organisms (so far used as a drug carrier in intranasal vaccines) [24].

### 3.4. The Risk of Intranasal Synechiae and the Intranasal Material Used

Scientists investigated the relationship between the incidence of intranasal adhesions after nasal septum surgery (Cottle’s mod. septoplasty) and the use of tamponade. The population was divided into three groups: the control group (without using a nasal dressing), “+ Telfa”—study group 1 (conventional [gauze seton] material was used) and “Merocel alone”—study group 2 (Merocel was used as a dressing). The study showed no statistically significant differences in the incidence of intranasal adhesions within 2 weeks of surgery [25].

Dutta et al. compared (in terms of effectiveness, costs, and complications) the use of a traditional gauze seton, Merocel and a modification of a conventional gauze nasal dressing (a piece of aluminum foil, obtained from the suture material cover). The purpose of this modification was to better stabilize and secure the nasal septum and consequently to contribute to reducing the risk of damage to the nasal mucosa. It has been shown that the use of aluminum foil (as a septum splint, with the addition of conventional seton gauze) can reduce the frequency of mucosal damage, the formation of intranasal adhesions, and also has a satisfactory hemostatic effect [26].

### 3.5. Use of Intranasal Dressings Based on Absorbed Materials

The hypothesis regarding the redundancy of dressing materials after operations within the nasal cavities is debatable. Most surgeons share the view that the nasal dressing is necessary. The use of dressing material in patients with diagnosed arterial hypertension, diabetes, and inflammatory reactions is obvious and leaves no doubts [27].

A meta-analysis of four studies did not show the advantage of fibrin tissue adhesive (FTA) over traditional packing of the nasal cavities after rhinosurgical treatment [28].

Another study analyzed the clinical benefits of using an absorbable material (NasoPore) impregnated with a steroid (betamethasone), an antibiotic (ciprofloxacin), and saline (control group). There were no statistically significant differences between the above cohorts in the formation of intranasal adhesions during the 180-day follow-up period [29].

One study in an animal model (sheep) showed a reduction in adhesion formation after FESS using a chitosan gel-releasing dressing. Additionally, microporous polysaccharide hemispheres effectively reduced postoperative bleeding during the recovery period after FESS [30].

Meta-analyses compared the use of intranasal dressings based on non-absorbable materials (Merocel) with absorbable materials (NasoPore). The evaluated criteria were pain, feeling of expansion in the nasal cavity, general satisfaction, feeling of nasal obstruction, and healing process. The results revealed no statistically significant differences between the two groups in the formation of intranasal adhesions. The general conclusion, however, was in favor of the use of NasoPore [27,31,32,33].

Research has also been conducted in the context of the selection of the most effective absorbable dressing. A multicenter study comparing gelatin sponge-based materials (Cutanplast and Spongostan) after FESS showed no significant differences in postoperative bleeding control. The advantage of Cutanplast (resulting from its unique structure—larger volume of gelatin [677 mg], higher porosity, and smaller volume of pores in the sponge) was thought to be its easier and less painful application, faster wound healing, and economic benefits. There were no differences in the formation of intranasal adhesions [34].

#### Prevention of Lateralization of the Middle Turbinates after Functional Surgery of the Paranasal Sinuses (FESS)

The use of maneuvers preventing excessive lateralization of the middle turbinates during FESS reduced the risk of intranasal adhesions [35].

### 3.6. Surgical Approaches and the Risk of Intranasal Adhesions

The surgical technique used also has an impact on the incidence of adhesions. Based on a review of the literature (of randomized prospective studies), it was shown that performing septal surgery using the classical method (e.g., the Cottle, or Killian approaches) results in a statistically significantly higher incidence of intranasal adhesions compared to endoscopic surgery [36].

### 3.7. Application of Mitomycin C

A risk factor for the formation of intranasal fusion after nasal septum surgery is damage to the mucoperichondral lobe, especially in the case of simultaneous surgery with the lateral wall of the nose (e.g., conchoplasty). The solution to this complication may be the addition of 1 mL of mitomycin C (a cytostatic drug used in cancer therapy, showing anti-proliferative effects against fibroblasts), at a concentration of 0.4 mg/mL, placed on the swabs for about 5 min. Then, the operating area should be irrigated with about 30 mL of distilled water. The authors observed a statistically significant reduction in the incidence of adhesions compared to the control group. Although the study was conducted on a small group, it seems to be an interesting direction in the prevention of adhesions, especially in patients with significant risk factors [37].

However, a similar relationship was not observed in patients undergoing functional surgery of the paranasal sinuses (FESS) [38].

### 3.8. Intranasal Synechiae in Other Types of Surgery with the Use of Intranasal Approach

One study analyzed the effects of lacrimal sac surgery (dacryocystorhinostomy, DCR), and showed that intranasal synechiae occurred in 9.6% of patients. The means to reduce the frequency of adhesions were not the subject of the study [39].

In the case of cranial base surgery with the creation of a nasoseptal flap (performed for various reasons, e.g., pituitary adenomas, benign and malignant tumors, etc.), the risk of intranasal adhesions was estimated at 19.5% [40].

## 4. Discussion

The nasal cavity is the first part of the upper respiratory tract. It has multiple functions: respiratory, filtration (air purification), thermal, and sensual (vomeronasal organ). It affects the formation of respiratory tract resistance, being responsible for about 30–50% of the total resistance exerted by the inhaled air.

Histologically, the nasal mucosa is composed of ciliated squamous epithelial tissue. In addition, the tissue consists of basal, goblet and striated cells. Damage to the function of the cilia, and deterioration of mucus production by glandular cells, lead to disorders of homeostasis. Tissue infiltration by inflammatory cells (eosinophils, neutrophils, plasma cells, and lymphocytes), and abnormal mucociliary clearance, generate common pathologies of the respiratory tract, including rhinitis and rhinosinusitis [3,41].

Intranasal adhesions called synechiae (labeled, according to the ICD-10, as J34.8—other specified diseases of the nose and paranasal sinuses) are pathologies that occur as a result of improper healing of the damaged nasal mucosa. Any actions in the nasal cavity and the lateral wall of the nose (including nasal tamponade and surgical treatment) lead to fluctuations in the cell–cytokine balance. Fibroblasts and myofibroblasts (activated via TGF-β and transforming growth factor β), directly (through the production of collagen) contribute to the development of intranasal adhesions [42].

Synechiae are usually the result of iatrogenic effects occurring as a result of rhinosurgical treatment. Less often, they occur as complications of neurosurgical operations (e.g., removal of pituitary tumors by intranasal approach). Injuries (foreign bodies, battery, contact sports, and accidents) are also important [43]. Intranasal adhesions may also be the result of prolonged local inflammation, generalized diseases of blood vessels, necrosis and vasculitis, and even in Sjögren’s syndrome [44]. Intranasal adhesions may also be accompanied by synechia of other parts of the upper respiratory tract, e.g., the nasopharynx [45]. So far, no universal classification system for intranasal adhesions has been developed, but a certain proposal (depending on the etiology) was put forward by Alova et al. (Table 1) [46].

The most popular materials used as an anterior tamponade include bandage gauze, paraffin, Vaseline, bismuth iodoform paraffin paste, glove fingers, Silastic, gelfoam, merocel, gauze impregnated with various antibiotics, fibrin glue, and absorbable materials, as well as pneumatic tools (such as balloons or Foley catheters) [16,47,48]. 

Tamponing of the nasal cavity, like any procedure, carries the risk of complications, e.g., damage to the mucous membrane and perforation of the septum, breathing disorders during sleep, decreased arterial oxygen saturation (during sleep), movement and suction of material, allergy, toxic shock syndrome, eustachian tube dysfunction, otitis media with effusion, and paraffin-induced granuloma. They cause discomfort, pain and sometimes lead to bleeding [10,49]. Experimental studies show that the use of nasal tamponade increases discomfort and pain in the postoperative period, and damages the nasal mucosa, including reducing the motility of the cilia of the ciliary epithelium [50,51]. No difference in postoperative complications (especially hemorrhagic or septal hematoma) in the absence of anterior nasal tamponade after nasal septum surgery was found. A possible alternative to packing the nasal cavity (especially with bandage gauze) is to make a trans-septal (nasal septum) suture. This action, which does not require packing the nasal cavities, reduces the feeling of postoperative pain and discomfort [52].

One of the most commonly used insoluble dressing materials (other than bandage tamponade) is Merocel (Medtronic Inc., Minneapolis, MN, USA), which is a compressed, dehydrated sponge composed of hydroxylated polyvinyl acetate. Expansion occurs under the influence of moisture (e.g., blood, mucosal secretions, or saline). Merocel, by increasing its volume, exerts a mechanical effect (physical pressure), causing obstruction of the bleeding blood vessels of the nasal cavity, which promotes a hemostatic effect [53]. The downside of using Merocel is the unpleasant pain that occurs during the removal of the dressing. Other complications have also been reported, such as dislocation of the dressing, perforation of the hole in the nasal septum, obstructive sleep apnea, and even septic shock syndrome and death [54]. 

If the use of an intranasal dressing (especially an insoluble one) is not possible, the ideal solution would be to use a material that fully inhibits bleeding and at the same time minimizes unpleasant sensations for the patient—pain and discomfort—and does not negatively affect the nasal mucosa and cause the occurrence of intranasal adhesions.

Currently, dissolving materials have become an alternative, which are reported to effectively stop bleeding and do not require removal of the dressing which causes pain and discomfort. An example is the commonly used NasoPore (Polyganics, Groningen, the Netherlands)—a synthetic, biodegradable foam produced in the lyophilization process. During the absorption of water (e.g., from blood), it has a hemostatic effect, exerting mechanical pressure on blood vessels, and also mechanically supports the surrounding tissue. The process of dissolving the foam occurs within a few days, which allows for relatively painless aspiration from the lumen of the nasal cavity [55]. In terms of the disadvantages of the use of NasoPore, a study was published which reports excessive process of granulation within the postoperative wound [31]. 

Gelatin-based absorbable materials have been used in clinical practice since 1945. Gelfoam (Pharmacia & Upjohn Company, New York, NY, USA) and the popular Spongostan (Ferrosan, Copenhagen, Denmark) are absorbable (water-insoluble) gelatin sponges. They are obtained from the purified gelatin of pork skins. Cutanplast (Mascia Brunelli S.p.A., Milan, Italy) is a new sterile, absorbable gelatin sponge with a strong hemostatic effect. What distinguishes Cutanplast from Spongostan is the structure (porosity and pore volume) of the sponge due to the density of gelatin. This modification results in a more efficient production of the hemostatic effect. However, studies have not shown differences in the formation of intranasal adhesions [34,56,57]. 

Reports also mention the use of cytostatic drugs in the primary prevention of intranasal adhesions in carefully selected cases. Mitomycin C is an anticancer drug, first obtained in 1958 from the Streptomyces caespitosus strain. It works by inhibiting DNA synthesis through bifunctional DNA alkylation, which prevents cell proliferation. Used in higher doses, it affects the synthesis of RNA and proteins. Topical use inhibits scar formation [58,59].

### 4.1. Surgical Technique, Additional Maneuvers and the Risk of Adhesions

Surgical technique also has a significant impact. Endoscopic surgery of the nasal septum improves the insight into the operating field (especially the posterior and lower sections), and shortens the procedure time. The rate of intra-operative and postoperative complications (including adhesions) is lower in the case of endoscopic procedures. The possibility of teaching and supervising the work of less experienced surgeons also favors endoscopic operations [35]. 

The increased incidence of intranasal adhesions also includes excessive lateralization (moving towards the lateral wall of the nasal cavity) of the middle turbinate during FESS. The literature mentions several methods of preventing excessive lateralization—the most popular ones are described in Table 2 [36,60].

### 4.2. Care in the Postoperative Period

Proper care of the nasal mucosa in the postoperative period and a postoperative follow-up are indisputably necessary. In addition to the importance of irrigation, the correct technique is important, as well as the use of additional substances in appropriate concentrations, e.g., sodium chloride or hyaluronan. At the same time, hyaluronic acid is a factor influencing the homeostasis of the epithelium of the respiratory tract (lower and upper), as well as a significantly important component of the extracellular substance [22].

### 4.3. Limitations

The main limitations of this study are the reliance on open access articles in public databases. Secondly, studies and meta-analyses were considered only in English. Case studies were also excluded from the study, but despite their relatively low evidentiary quality, they are sometimes a signpost for unconventional cases.

Given the above, we are aware that the above criteria may limit our work.

## 5. Conclusions

1. Irrigation of the nasal cavities in the postoperative period significantly reduces the risk of intranasal adhesions after rhinosurgical treatment. The type and concentration of the solution used during irrigation is also important.
2. The use of absorbable materials reduces the risk of intranasal adhesions.
3. The use of splints after turbinoplasty clinically significantly reduces the risk of intranasal adhesions.
4. The use of splints made of modern materials (e.g., Silastic) after nasal septum surgery and endoscopic surgery of the paranasal sinuses may reduce the risk of intranasal adhesions.
5. In carefully selected cases, as a preventive factor or in patients not responding to standard methods, local cytostatic treatment (mitomycin C) should be used.
6. The risk of intranasal adhesions is reduced by factors related to the operation itself: simultaneous operation of the inferior turbinates, endoscopic septoplasty, and protection of the middle turbinate against excessive lateralization during FESS.
7. An option to reduce the risk of intranasal adhesions after septoplasty is suturing of the nasal septum.
8. Further research is required that may result in a universal classification system for intranasal adhesions that could be used as a preventive measure, and on the other hand, facilitate individualization of the therapy (e.g., selection of the appropriate form of treatment depending on the stage of advancement).
9. Summarizing—the risk of intranasal adhesions (synechiae) depends on the type of procedure, the surgical technique, materials used and wound care in the postoperative period. Every case requires an individual approach.

## Figures and Tables

**Table 1:** Causes of damage to the mucous membrane of the nasal cavity leading to the formation of intranasal adhesions, according to Alova et.al. [46].

Cause	Examples of Diseases and Circumstances
Infectious	Rhinoscleroma, Rhinosporidiosis, Leishmaniasis. Other: Mycobacteria (*M. tuberculosis*, *M. leprae*), Syphilis.
Autoimmune and non-infectious	Granulomatosis with polyangiitis, Cicatricial pemphigoid, Epidermolysis bullosa acquisita, Sarcoidosis, Sjögren’s syndrome.
Traumatic	Accidental (including contact sports, foreign body); Iatrogenic (surgery, packing, etc.).
Others	Intranasal drugs (cocaine), physical (radiotherapy) and chemical burns Radiotherapy, Natural Killer/T cell lymphoma-nasal type, Intranasal eosinophilic angiocentric fibrosis.

**Table 2:** Ways to prevent excessive lateralization of the middle turbinate during functional endoscopic paranasal sinus surgery (FESS) [36,60].

Method	Characteristics	Comments
Bolgerization	Controlled formation of turbinate–septal adhesion.	Named after the propagator of the method, Bolger.
Conchopexy	Connection (through the nasal septum with absorbable surgical suture, size 4–0) of the heads of the middle turbinates.	The most effective method of preventing adhesions (according to many surgeons).
Clipping	Fixation of the middle turbinate heads to the septum using metal clips.	This method is not recommended in the case of polypus degeneration of the nasal turbinates or previous (submucosal) resection of the nasal turbinate and the absence of septal cartilage (prevention of iatrogenic septal perforation).
Partial turbinate resection	Partial/complete resection of the middle turbinate.	The consequence may be iatrogenic obliteration of the frontal sinus.
Implantation of a steroid-eluting stent	Implantation of an absorbable steroid-eluting stent, e.g., mometasone furoate.	In addition (to the mechanical effect, that fixes the turbinate in the correct position) it also has an anti-inflammatory and anti-edematous effect, positively affecting the healing process of the mucous membrane.

## Data Availability

Not applicable.

## Supplementary Materials

The following supporting information can be downloaded at: https://www.mdpi.com/article/10.3390/jcm12216831/s1, Table S1: summarizing the characteristics of the included studies.
