# Supplementary material for: Intranasal Synechiae as Complications of Rhinosurgical Treatment—A Review of Current Knowledge
Source: J Clin Med. 2023 Oct 29;12(21):6831. doi: 10.3390/jcm12216831 (PMC10648208; doi:10.3390/jcm12216831)
Supplement: Supplementary file 1 [file jcm-12-06831-s001.zip › jcm-2625102-supplementary.pdf]

Journal of
Clinical Medicine

MDPI

Supplement—Table S1. summarizing the characteristics of the included studies.

Number	Title	Author(s)	Source	Comments
**1.**	Deviated nose: Physiological and pathological changes of the nasal cavity.	Kim TK, Jeong JY	*Arch Plast Surg. 2020 Nov;47(6), pp. 505-515.*	Pathway
**2.**	Synechiae in the Nose treated by Diathermy	McKenzie D	*Proc R Soc Med. 1915; 8 (Laryngol Sect), p 74.*	First article about nasal adhesion treatment
**3.**	History of intranasal splints	Lau, J., Elhassan, H., Singh, N	*The Journal of Laryngology & Otology, 2018. 132(3), pp. 198-201.*	Role of splints
**4.**	The value of intranasal splints after partial inferior turbinectomy	Awad OG, Hamid KA.	*Indian J Otolaryngol Head Neck Surg. 2015 Mar;67(1), pp. 75-80.*	Role of splints
**5.**	Should intranasal splints be used after nasal septal surgery?	Tang S, Kacker A.	*Laryngoscope. 2012 Aug;122(8), pp.1647-8.*	Role of splints
**6.**	Objective usefulness of thin silastic septal splints after septal surgery.	Jung YG, Hong JW, Eun YG, Kim MG.	*Am J Rhinol Allergy 2011;25, pp. 182–185.*	Role of splints
**7.**	Intranasal splints and their effects on intranasal adhesions and septal stability.	Cook JA, Murrant NJ, Evans KL, Lavelle RJ.	*Clin Otolaryngol Allied Sci. 1992 Feb;17(1), pp. 24-7.*	Role of splints
**8.**	Nasal septal clips: an alternative to nasal packing after septal surgery?	Veluswamy A, Handa S, Shivaswamy S.	*Indian J Otolaryngol Head Neck Surg. 2012 Dec;64(4), pp. 346-50.*	Role of splints
**9.**	Silastic splints reduce middle meatal adhesions after endoscopic sinus surgery.	Baguley CJ, Stow NW, Weitzel EK, Douglas RG.	*Am J Rhinol Allergy. 2012 Sep-Oct;26(5), pp. 414-7.*	Role of splints
**10.**	Silastic "Spring" Spacers for Use Following Endoscopic Sinus Surgery.	Hartl TT, Ospina J, Janjua A.	*Indian J Otolaryngol Head Neck Surg. 2019 Jun;71(2), pp. 233-237.*	Role of splints

Number	Title	Author(s)	Source	Comments
**11.**	Suturing of the nasal septum after septoplasty, is it an effective alternative to nasal packing?	Al-Raggad DK, El-Jundi AM, Al-Momani OS, Al-Serhan MM, Nawasrah OO, Qhawi MA, Husban AM.	*Saudi Med J 2007;28(10), pp. 1534-6.*	Role of suturing
**12.**	Comparison of suture and nasal packing in rabbit noses.	Genç E, Ergin NT, Bilezikçi B.	*Laryngoscope 2004;114(4), pp. 639-45.*	Role of suturing
**13.**	Septal suturing following nasal septoplasty, a valid alternative for nasal packing?	Lemmens W, Lemkens P.	*Acta Otorhinolaryngol Belg 2001;55(3), pp. 215-21.*	Role of suturing
**14.**	A novel way of trans-septal splint suturing without nasal packing for septoplasty.	Naik K.	*Indian J Otolaryngol Head Neck Surg. 2015 Mar;67(1), pp. 48-50.*	Role of suturing
**15.**	Retrospective analysis of 697 septoplasty surgery cases: packing versus trans-septal suturing method.	Cukurova I, Cetinkaya EA, Mercan GC, Demirhan E, Gumussoy M.	*Acta Otorhinolaryngol Ital. 2012 Apr;32(2), pp. 111-4.*	Role of suturing and dressing
**16.**	Comparison of Trans-septal Suturing Technique With Polyvinyl Alcohol Sponge-Based Nasal Packing for Hemostasis in Septoplasty.	Meena R, Sharma R, Malhotra V, Rathore PK.	*Cureus. 2022 May 20;14(5), p. e25161.*	Nasal dressing
**17.**	Mucociliary clearance and buffered hypertonic saline solution.	Talbot AR, Herr TM, Parsons DS.	*Laryngoscope. 1997;107, pp. 500-3.*	Postoperative care, wound care.
**18.**	Effects of buffered saline solution on nasal mucociliary clearance and nasal airway patency.	Keojampa BK, Nguyen MH, Ryan MW.	*Otolaryngol Head Neck Surg. 2004;131, pp. 679-82.*	Postoperative care, wound care.
**19.**	The effect of different nasal irrigation solutions following septoplasty and concha radiofrequency: a prospective randomized study.	Kurtaran H, Ugur KS, Yilmaz CS, Kaya M, Yuksel A, Ark N, Gunduz M.	*Braz J Otorhinolaryngol. 2018 Mar-Apr;84(2), pp. 185-190.*	Postoperative care, wound care.
**20.**	Hyaluronan plus saline nasal washes in the treatment of rhino-sinusal symptoms in patients undergoing functional endoscopic sinus surgery for rhino-sinusal remodeling.	Macchi A, Terranova P, Digilio E, Castelnuovo P.	*Int J Immunopathol Pharmacol. 2013 Jan-Mar;26(1), pp. 137-45.*	Postoperative care, wound care.
**21.**	Anti-adhesive effect of solid mixture of sodium hyaluronate/carboxymethylcellulose in murine nasal cavities.	Lee EJ, Hwang HJ, Jung CM, Kim MK, Kim KS.	*Eur Arch Otorhinolaryngol. 2017 Jan;274(1), pp. 181-188.*	Postoperative care, wound care.

Number	Title	Author(s)	Source	Comments
**22.**	Development and Optimization of Chitosan Nano-particle-Based Intranasal Vaccine Carrier.	Gao, X.; Liu, N.; Wang, Z.; Gao, J.; Zhang, H.; Li, M.; Du, Y.; Gao, X.; Zheng, A.	*Molecules 2022, 27, p. 204.*	Postoperative care, wound care.
**23.**	A comparative study on nasal packing after septo-plasty: does it matter in terms of patient comfort, bleeding, and crust or synechia formation?	Özbal Koç AE, Türkoğlu Baba-kurban S, Kibar SS, Büyüklü F.	*Kulak Burun Bogaz Ihtis Derg. 2016 May-Jun;26(3), pp. 152-8.*	Nasal dressing
**24.**	Modified technique of anterior nasal packing: a comparative study report.	Dutta S, Mukherjee A, Saha J, Biswas G, Haldar D, Sen I, Sinha R.	*Indian J Otolaryngol Head Neck Surg. 2012 Dec;64(4), pp. 341-5.*	Nasal dressing
**25.**	Merocel versus Nasopore for nasal packing: a meta-analysis of randomized controlled trials.	Wang J, Cai C, Wang S.	*PLoS One. 2014 Apr 7;9(4), p. e93959.*	Nasal dressing
**26.**	Fibrin tissue adhesive versus nasal packing in en-doscopic nasal surgery: a systematic review and meta-analysis.	Coey JG, Whittaker PJ, Williams G, Ikram UH, Page OJR.	*Rhinology. 2019 Feb 1;57(1), pp. 21-31.*	Nasal dressing
**27.**	Steroid vs. antibiotic impregnated absorbable nasal packing for wound healing after endoscopic sinus surgery: a randomized, double blind, placebo-con-trolled study.	Grzeskowiak B, Wierzchowska M, Walorek R, Seredyka-Burduk M, Wawrzyniak K, Burduk PK.	*Braz J Otorhinolaryngol. 2019 Jul-Aug;85(4), pp. 473-480.*	Nasal dressing
**28.**	Effects of a novel chitosan gel on mucosal wound healing following endoscopic sinus surgery in a sheep model of chronic rhinosinusitis.	Athanasiadis T, Beule AG, Rob-inson BH, Robinson SR, Shi Z, et al.	*The Laryngoscope 118, pp. 1088–1094.*	Nasal dressing
**29.**	The effects of Vaseline gauze strip, Merocel, and Nasopore on the formation of synechiae and exces-sive granulation tissue in the middle meatus and the incidence of major postoperative bleeding after endoscopic sinus surgery.	Wang YP, Wang MC, Chen YC, Leu YS, Lin HC, Lee KS.	*J Chin Med Assoc. 2011 Jan;74(1), pp. 16-21.*	Nasal dressing
**30.**	Absorbable and nonabsorbable packing after func-tional endoscopic sinus surgery: systematic review and meta-analysis of outcomes.	Wang TC, Tai CJ, Tsou YA, Tsai LT, Li YF, Tsai MH.	*Eur Arch Otorhinolaryngol. 2015 Aug;272(8), pp. 1825-31.*	Nasal dressing
**31.**	Comparative analysis of Cutanplast and Spon-gostan nasal packing after endoscopic sinus sur-gery: a prospective, randomized, multicenter study.	Cho KS, Park CH, Hong SL, Kim MJ, Kim JY, Kim YW, Koo SK, Roh HJ.	*Eur Arch Otorhinolaryngol. 2015 Jul;272(7), pp. 1699-705.*	Nasal dressing

Number	Title	Author(s)	Source	Comments
**32.**	Endoscopic vs. conventional septoplasty: A review of the literature.	Champagne C, Ballivet de Régloix S, Genestier L, Crambert A, Maurin O, Pons Y.	*Eur Ann Otorhinolaryngol Head Neck Dis. 2016 Feb;133(1), pp. 43-6.*	Other types of septoplasty
**33.**	Effect of middle turbinate intervention on outcomes of middle meatal endoscopic surgery	Bofares KM.	*Int J Otorhinolaryngol. 2015, 1, pp. 13-19*	Additional maneuvers during surgery
**34.**	Preventive Measures of Middle Turbinate Lateralization After Endoscopic Sinus Surgery: An Updated Review.	Alaryani RA, Alhedaithy RA.	*Cureus. 2021 Jun 19;13(6), p. e15763.*	Additional maneuvers during surgery
**35.**	The use of Mitomycin-C to reduce synechia in middle meatus in sinus surgery: preliminary results.	Yamaoka WY, Gregório LC.	*Braz J Otorhinolaryngol. 2012 Oct;78(5), pp. 44-50.*	Other methods of preventing adhesions
**36.**	Results of endoscopic endonasal dacrocystorhinostomy.	Aslam MA, Mirza AB, Butt IA.	*J Pak Med Assoc. 2014 Jun;64(6), pp. 619-23.*	Other types of surgery leading to intranasal adhesions
